# Higher levels of VEGF-A and TNFα in patients with immune checkpoint inhibitor-induced inflammatory arthritis

**DOI:** 10.1186/s13075-025-03546-3

**Published:** 2025-04-01

**Authors:** Elise F. Gray-Gaillard, Ami A. Shah, Clifton O. Bingham III, Jennifer H. Elisseeff, Joseph Murray, Julie Brahmer, Patrick Forde, Valsamo Anagnostou, Jennifer Mammen, Laura C. Cappelli

**Affiliations:** 1 Johns Hopkins School of Medicine, Bloomberg Kimmel Institute for Cancer Immunotherapy, Baltimore, MD USA; 2 Division of Rheumatology, Johns Hopkins School of Medicine, 5501 Hopkins Bayview Circle Suite 1B1, Baltimore, MD 21224 USA; 3 Department of Oncology, Johns Hopkins School of Medicine, Baltimore, MD USA; 4 Division of Endocrinology, Johns Hopkins School of Medicine, Baltimore, MD USA

## Abstract

**Background:**

Immune checkpoint inhibitors (ICI), a type of cancer immunotherapy, can cause side effects including inflammatory arthritis (ICI-IA). Previous studies of ICI-IA do not include a thorough characterization of associated immune responses to provide potential targets for treatment. We aimed to identify cytokines uniquely increased in ICI-IA and determine correlations with IA severity and persistence.

**Methods:**

We evaluated patients diagnosed with ICI-IA by a rheumatologist (*n* = 80); control serum was obtained from ICI-treated cancer patients without any diagnosed irAEs (*n* = 17) or diagnosed with an unrelated irAE (*n* = 19). Serum was assayed to quantify 9 cytokine levels (IFN-γ, IL-4, IL-6, IL-10, IL-12p70, IL-1α, TNF-α, IL-17a, VEGF-A) using MSD U-PLEX assay. Mann-Whitney U tests were performed to evaluate differences in cytokine levels between control and ICI-IA groups. The Kruskal-Wallis test and multivariable ordinal logistic regression were used to determine difference in cytokine levels between patients of differing disease activity.

**Results:**

VEGF-A and TNFα were significantly elevated in patients with ICI-IA compared to ICI-controls; results persisted when restricting analyses to patients not treated with immunosuppressants at the time of sampling. ICI-IA patients were stratified by IA severity using CDAI score; there was significantly higher VEGF-A in those with higher disease activity. Ordinal logistic regression showed higher levels of IL-6 and VEGF-A were associated with higher disease activity.

**Conclusion:**

Elevated levels of VEGF-A and TNFα are associated with ICI-IA. There was also higher IL-6 and VEGF-A among those with higher disease activity when controlling for confounding. These cytokines could be used as biomarkers of ICI-IA severity and present therapeutic targets.

**Supplementary Information:**

The online version contains supplementary material available at 10.1186/s13075-025-03546-3.

## Introduction

The discovery of checkpoint molecules and development of antibodies to block them, immune checkpoint inhibitors (ICIs), have revolutionized cancer therapies [1]. Since the first approval of ICIs for melanoma in 2011, ICIs have shown unprecedented clinical responses and are being implemented as the standard of care for an increasing number of advanced malignancies [2–7]. As a result, an increasing number of immunotherapies are being developed and clinically approved to target checkpoint molecules. There are currently 13 FDA-approved agents targeting programmed cell death protein-1 (PD-1), programmed death ligand-1 (PD-L1), cytotoxic T-lymphocyte associated protein-4 (CTLA-4), or lymphocyte activation gene 3 (LAG-3).

Due to the potent nature of immune activation from ICIs, however, some patients experience inflammation and damage to non-tumor tissues, referred to as immune related adverse events (irAEs). These events arise since ICIs interrupt natural immune homeostasis by impeding immune tolerance of self antigens [8, 9]. IrAEs have been observed in around 34% of cancer patients treated with ICIs, and can affect a variety of tissue types [10]. Dermatitis, pneumonitis, and colitis are the most common forms of irAEs, and the latter two can be life-threatening [11]. Even non-life threatening irAEs can greatly impair the quality of life of patients, and some irAEs can persist for months to years [12]. While there are guidelines from the American Society of Clinical Oncology and the European Society of Clinical Oncology for the identification and management of acute irAEs, there are few guidelines for the management of chronic irAEs. Treating chronic irAEs can lead to further complications due to prolonged immunosuppression. Therefore, due to the increased use of ICIs in the (neo)adjuvant settings, we need to better understand chronic irAEs.

ICI-induced inflammatory arthritis (ICI-IA) is the most common irAE encountered by rheumatologists, can persist after ICI cessation [13, 14], and cause permanent joint damage [15, 16]. ICI-IA has been estimated to develop in 3-7.5% of patients treated with ICIs, while up to 43% of patients treated with immunotherapies have reported arthralgias [8]. ICI-IA often requires immunosuppression with corticosteroids or other therapies like tumor necrosis factor (TNF)-inhibitors and interleukin (IL)-6R inhibitors. Currently, treatment is empiric and not guided by prospective data or translational findings. A retrospective multicenter observational study suggested that TNF-inhibitors may be more effective for treatment of ICI-IA, but there was a trend toward increased cancer progression [17]. It is unclear which type of immunosuppression is the most effective for ICI-IA and least likely to affect the anti-tumor immune response.

While other studies have evaluated how ICI regimen impacts IA severity and persistence, previously published studies of ICI-IA do not include a thorough characterization of associated immune responses to provide potential treatment targets. Other forms of arthritis involve several proinflammatory cytokines that contribute to joint inflammation and cartilage and bone erosion, such as TNF-α, IL-1, IL-6, IL-17α, IL-12, granulocyte/macrophage colony-stimulating factor (GM-CSF), interferon-gamma (IFNγ), and vascular endothelial growth factor (VEGF) [18–20]. Conversely, some anti-inflammatory cytokines, such as IL-4 and IL-10, can decrease or resolve immune activity in inflammatory arthritis leading to decreased joint inflammation and pain [21]. With this study, we sought to identify soluble factors that are associated with ICI-IA and determine how those factors associate with IA severity and persistence.

## Methods

### Patients

Patients with ICI-IA from a longitudinal cohort study of rheumatic irAEs were included if they had rheumatologist diagnosed ICI-IA and a serum sample available from their baseline rheumatology visit where ICI-IA was diagnosed. Serum was obtained at a range of times post-ICI-IA symptom onset varying between 1.7 weeks- 57 months (median: 4 months). Control patients were patients with lung cancer treated with ICI therapy (anti-PD-1/PD-L1 monotherapy or combination ICI therapy); some had no irAEs (*n* = 17) and others had an irAE besides ICI-IA (*n* = 19). Serum was obtained from control patients at various times after the start of ICI treatment (control no-irAE median: 4.14 months [0 weeks, 27.4 months]; control other-irAE median: 5.28 months [9 weeks, 34.7 months]). This study was approved by the Johns Hopkins University Institutional Review Board (IRB00263113).

### Clinical measures

Baseline data including demographics, cancer type, ICI treatment, other irAEs, laboratory studies (anti-cyclic citrullinated peptide antibodies (CCP), rheumatoid factor (RF), antinuclear antibody (ANA)), clinical examination findings (tenosynovitis, enthesitis), and any treatments given due to IA such as steroids, conventional synthetic disease-modifying antirheumatic drugs (csDMARDS, e.g. methotrexate), or biologic (b)DMARDS (e.g. infliximab, adalimumab, and etanercept) were obtained at the initial visit. Follow up information on ICI-IA severity, quantified by clinical disease activity index (CDAI) score, and IA persistence was obtained for up to 71 months post initial visit.

### Serum soluble factor quantification

Cytokines (IFN-γ, IL-4, IL-6, IL-10, IL-12p70, IL-1α, TNF-α, IL-17a, VEGF-A) were analyzed in serum using electro-chemiluminescent labels (MSD GOLD SULFO-TAG) on U-PLEX assay platform by Meso Scale Discovery (MSD, Gaithersburg, MD). Three kits were used to analyze 120 serum samples in duplicate: U-PLEX Biomarker Group 1 (hu) Assays, SECTOR (MSD, catalog #K15067L-1), read on instrument QuickPlex SQ120 (MSD).

### Statistics

Patient data was stratified based on treatment with biologics or steroids at time of blood draw. Cytokine level data was tested for normality using various normality tests: Shapiro-Wilk, Kolmogorov-Smirnov, Anderson-Darling, and D-Agostino & Pearson. As the data did not fit a normal distribution, non-parametric Mann-Whitney U tests were performed to determine whether there were statistically significance differences in cytokine levels (i) between control and ICI-IA groups and (ii) between those with and without IA persistence. The Kruskal-Wallis test was used to assess whether cytokine levels were significantly different amongst ICI-IA patients only with mild, moderate or severe disease activity as determined by CDAI. The Mann Whitney test was used to compare cytokine levels between patients with and without enthesitis and with and without tenosynovitis. Univariate and multivariable ordinal logistic regression models with the outcome of CDAI categories were used to evaluate the odds of higher disease activity as it relates to individual cytokine levels. Next, logistic regression was performed for the outcome of persistent ICI-IA at 6 months post ICI-cessation. Finally, cytokine levels were compared between patients who had arthritis improvement with TNF-inhibitor therapy and those who did not have an arthritis response using Mann-Whitney U tests.

## Results

### Baseline patient characteristics

Serum samples from 80 patients diagnosed with ICI-IA, 49 female and 31 male, were obtained between 1.7 weeks- 57 months post-ICI-IA symptom onset (Table 1). Of the 36 ICI-control patients, 50% were female, 17 did not have any irAEs and 19 had an unrelated irAE, such as pneumonitis, colitis, hepatitis, or thyroiditis. ICI-IA patients were monitored with a median follow-up of 16.2 months and average follow-up of 23 months (range 2.42 weeks-71 months). The median age for ICI-IA patients was 61.4 years, compared to 67 years for ICI-control patients (Table 1). The ICI-IA patients had a range of cancer types, the most prevalent being melanoma (35%) followed by lung cancer (25%) and gastrointestinal cancers (14%). Most of the ICI-control patients had NSCLC (94%), and 2 ICI-control patients had SCLC. Most patients were treated with ICI monotherapy targeting PD-1, PD-L1, or CTLA-4 (ICI-IA: 70%, ICI-control: 94%), while some were treated with combination ICI therapy (ICI-IA: 30%, ICI-control: 6%). Patients with ICI-IA, on average, had moderate levels (17.2 ± 11.1) of disease activity by CDAI (Table 1). Of the 80 patients with ICI-IA, 15 (19.2%) experienced tenosynovitis, while 23 (29.9%) had enthesitis. Some patients required treatment with csDMARDs (*n* = 29), biologics (*n* = 19), and/or steroids (*n* = 30) to reduce inflammation and alleviate pain. Regardless, likely due to the potency of ICI therapies, a majority of patients with ICI-IA (58 of 71) had persistent IA characterized by continued symptoms for greater than 6 months post ICI cessation. (Table 1).

### Serum cytokine concentrations and their association with ICI-IA

**Fig. 1:**
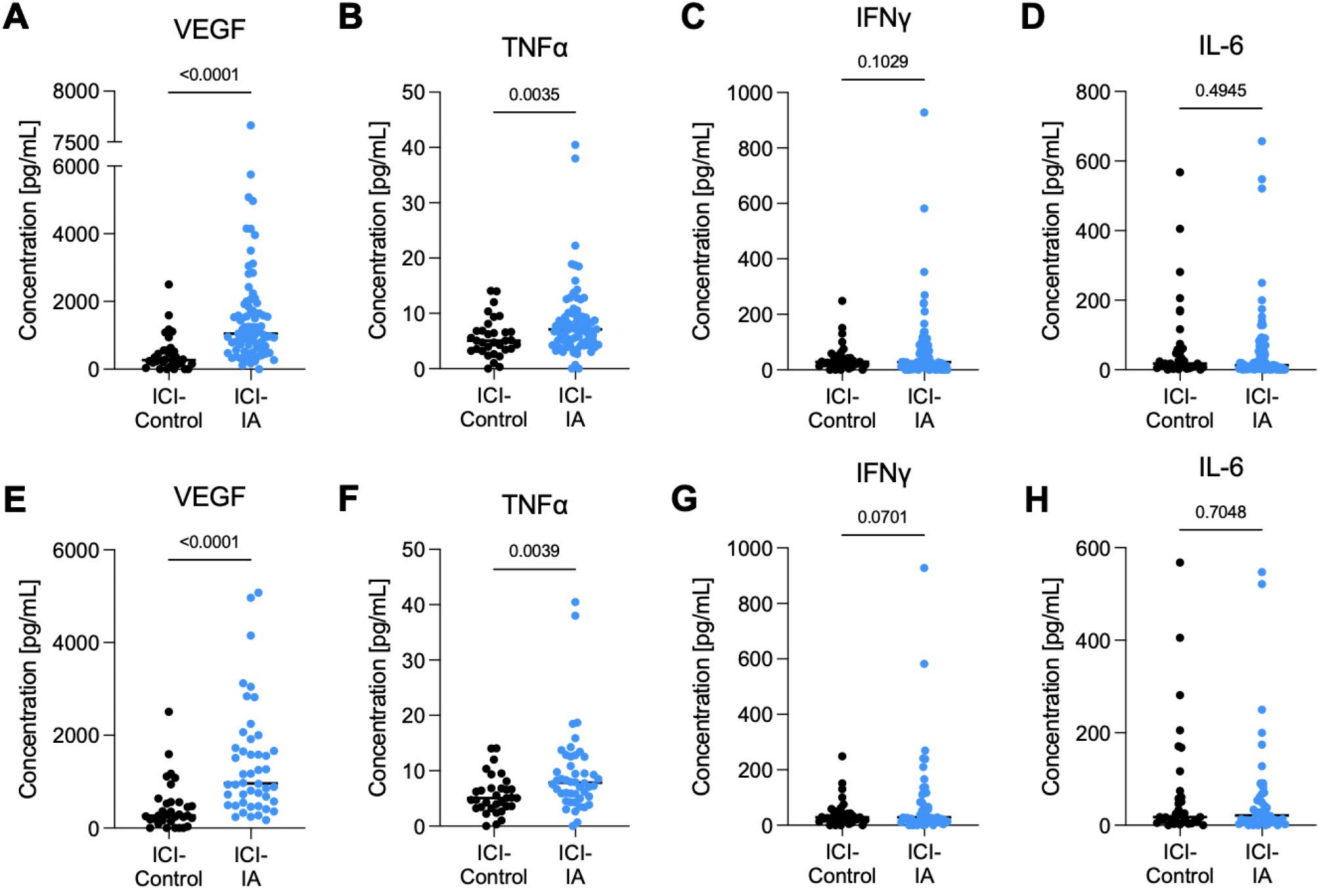
Serum cytokine levels of ICI-IA and ICI-Control patients. Abundance of (**A, E**) VEGF, (**B, F**) TNFα, (**C, G**) IFNγ, (**D, H**) IL-6 soluble factors in cancer patient serum treated with ICIs that developed ICI-IA (blue) versus patients treated with ICIs but did not develop ICI-IA (black). (**A-D**) All patients in this cohort with detectable levels of respective cytokines, and (**E-H**) patients without any prior treatment with steroids or biologics. Due to non-normal distribution of data, non-parametric Mann-Whitney U tests were run to determine significance

Serum from ICI-IA patients was sampled on average 37.2 months after the start of ICI treatment, while for ICI-control patients’ serum was obtained on average 18 months after ICI treatment commenced (Table 1). VEGF-A and TNFα were both significantly elevated in the serum of patients with ICI-IA compared to ICI-control (Fig. 1A and B). IFNγ and IL-6, common inflammatory cytokines associated with many pathologies, were not significantly different (Fig. 1C and D). Some ICI-IA patients were on steroids during the blood draw (37.5%) and/or required treatment with biologics (23.8%) for ICI-IA. As steroids and biologics (such as TNF-inhibitors) directly influence the immune system and could impact serum cytokine levels, we removed the ICI-IA patients treated with steroids or biologics (*n* = 33). Comparing the ICI-IA patients that were not treated with steroids or biologics (*n* = 47) to ICI-control patients (*n* = 36), VEGF-A and TNFα maintained significantly higher levels with IA (Fig. 1E-H). We validated that these cytokine changes were due to ICI-IA, not cancer type or ICI therapy, by comparing ICI-control and ICI-IA from NSCLC patients (A-D) and monotherapy (anti-PD1/PD-L1) (E-H) and observed increased VEGF-A and TNFα levels, consistent with Fig. 1 (Supplemental Fig. 1). To investigate whether the presence of an irAE within the ICI-control group would impact these cytokine levels we stratified the ICI-control patients by presence of irAE and found no differences in any of these cytokine levels (Supplemental Fig. 2). Further, no detectable differences were observed between ICI-IA and ICI-control for IL-10, IL-17α, IL-1α, IL-4, and IL-12p70 (Supplemental Table 1).

### Cytokine levels and IA clinical phenotype, severity, and persistence

Cytokine levels were compared between those who had enthesitis and those without. There was no significant difference in any of the cytokines between the groups (Supplemental Table 2). Cytokine levels were also compared in patients with ICI-IA who had tenosynovitis and those who did not. Again, no significant differences were found between the groups (Supplemental Table 2).

The severity of ICI-IA was determined with CDAI. The average CDAI (17.2) corresponded to moderate disease activity, with 25% of patients falling into a high IA severity, 44% moderate, and 26% low. To determine if the baseline cytokine levels correlated with IA severity, we stratified patients using their CDAI score. VEGF-A levels were higher in those with moderate or high disease activity (*p* = 0.02) while there were not significant differences in other cytokines by CDAI group (Table 2). Next ordinal logistic regression was used to evaluate cytokines where there were numeric trends toward higher levels in high disease activity in Table 2. There was a significantly elevated odds ratio for being in higher disease activity categories with increasing levels VEGF-A and IL-6 (Table 3). These odds ratios remained significant when controlling for age, sex, number of other irAEs, being on steroids at time of sample, and symptom duration before sample was taken (Table 3).

Of the 80 ICI-IA patients, 71 were monitored for more than 6 months to determine IA persistence. We defined persistence as continuation of joint pain and inflammation or inability to wean immunosuppression for ICI-IA for greater than 6 months after ICI cessation. Most patients (81.7%) had persistent IA. To determine if cytokine levels at initial IA diagnosis could predict IA persistence, we stratified the ICI-IA patients that were not on steroids at blood draw based on IA persistence and looked at the levels of all cytokines tested. We did not find any significant differences in baseline cytokine levels when comparing patients with and without persistent IA who were not on steroids at the time of the serum sample (Supplemental Table 3). Similarly, logistic regression did not show significant association between these cytokines and persistence (Supplemental Table 4).

### Cytokine levels and arthritis response to biologic therapy

We had 19 patients who received biologic or JAK-inhibitor therapy for ICI-IA; 18 received a TNF-inhibitor as the first biologic and one received tofacitinib. Nine of these patients were on steroids at the time of the blood draw for cytokine levels. Of the 18 patients treated with TNF-inhibitors first, one had previously received infliximab and had an infusion reaction to re-treatment so response could not be assessed, and another did not have follow up after starting therapy. Of the 16 remaining patients treated with TNF-inhibitors, 11 had improvement in symptoms with the TNF-inhibitor and five did not have improvement and needed to try other therapies. The median TNF level at baseline of responders was 8.0 pg/mL and for non-responders the median level was 5.1 pg/mL (*p* = 0.02, Table 4). Patients who had improvement of their ICI-IA with TNF-inhibitors also had higher IL-6 levels at baseline (53.1 pg/mL vs. 2.1 pg/mL, *p* = 0.03). Baseline VEGF levels did not differ significantly between those with and without arthritis response (Table 4).

## Discussion

With the increasing use of ICIs in the clinic for the treatment of cancer, more irAEs, including ICI-IA, are being diagnosed, often requiring immunosuppression and impacting patient quality of life. ICI-IA is not well understood and lacks biomarkers to predict its development or therapeutic targets to mitigate disease. This is among the first studies to quantify the systemic levels of arthritis-associated cytokines in ICI-IA patients and to correlate cytokine levels with phenotypic features and clinical outcomes. We found that elevated levels of VEGF-A and TNFα are associated with ICI-IA, regardless of steroid or biologics treatment. Furthermore, higher levels of baseline VEGF-A and IL-6 significantly correlated with increased IA severity. When solely looking at patients treated with biologics to treat their ICI-IA, we observed patients that responded well to anti-TNF therapy had significantly increased baseline TNFα and IL-6 levels, but not VEGF-A. This could indicate that these cytokines, VEGF-A and TNFα, via separate mechanisms, could be used as systemic biomarkers of ICI-IA and present possible treatment targets.

Prior translational studies in ICI-IA have focused on immune cell populations. Recent studies have focused on linking an increase in CD8^+^ cytotoxic T cells with the articular damage of ICI-IA, both in the joint, and systemically. One study identified a prominent presence of Th1-CD8^+^ T cells promoting ICI-IA and Th17-type skewing in response to ICI therapy [22]. Another found an increase in highly activated CD38^+^CD8^+^ T cells with IFNγ signatures, not only in joints, but also in the circulation [23]. Neither study profiled the secretome of other immune cells and other circulating soluble factors. The presence and level of peripheral cytokines has been widely used diagnostically, even in ICI-treated patients [24], to predict outcomes such as treatment efficacy, survival, and the development of irAEs.

Biomarkers measurable in the peripheral blood are particularly useful to guide clinical decision making due to the ease of access and limited invasiveness. Several studies have investigated the levels of serum cytokines in other forms of arthritis as well. Elevated levels of IL-6, IL-18, IFNγ, IL-17, IL-23, and TNFα have been found in the serum of patients with rheumatoid arthritis [25, 26], as well as psoriatic arthritis [27]. Not only have these serum cytokine analyses in other forms of arthritis identified biomarkers of disease, but they have also identified therapeutic targets for which therapies are being developed and assessed [26–28]. While it is unclear whether ICI-IA is an autoimmune disease as no autoantibodies have been found to be consistently associated with ICI-IA, it is still a systemic immune disease that can influence other tissues and the periphery [29]. Furthermore, as this form of IA is induced by ICI therapy, a potent systemic treatment, there is a clear need to explore the systemic cytokine changes that could correlate with ICI-IA as well as IA severity and persistence. Additionally, commercially available therapies exist to target VEGF, TNFα, and IL-6, enhancing the potential significance of these cytokines as treatment targets.

Cytokines in the peripheral blood have been evaluated in other forms of irAEs beyond ICI-IA. In one study Th17 associated cytokines, including IL-17 and IL-6, and TNF-alpha were increased early in ICI treatment in those with grade 2 or higher irAEs [30]. In this study, higher IL-6 levels were also associated with poor survival, further supporting IL-6 blockade as a rational treatment approach. In other studies, G-CSF or IL-17 levels at baseline were associated with future irAE development [31, 32]. Future studies of ICI-IA can also evaluate G-CSF as a candidate serum biomarker.

The study had several limitations. We had a large cohort of patients with ICI-IA and serum samples; however, increasing the patient cohort size and increasing to multiple collection sites would provide better insights into possible changes in cytokine levels across cancer types, ICI regimen, and IA severity and persistence. Additionally, not all baseline samples were taken at the exact same point in ICI-IA disease course. We tried to address this in within ICI-IA comparisons by controlling for symptom duration at the time of sample. While the ICI-IA patient cohort is representative of a diverse set of cancer types, we acknowledge the limited representation of cancer types in the ICI-control groups. For future studies, ICI-control patients across a greater variety of cancers should be evaluated. Further, while this study assayed the serum levels of nine cytokines specific to arthritis, it would be beneficial to screen the serum proteome in a deeper, un-biased way.

In conclusion, this study found that patients with ICI-IA had a higher pro-inflammatory and vascular response, specifically increased TNFα and VEGF-A. Furthermore, VEGF-A levels along with IL-6 increased with heightened IA severity. While these cytokines could be used as systemic biomarkers of ICI-IA, further proteomic profiling should be performed to quantify the levels of other pro-inflammatory and vascular-associated cytokines to identify additional therapeutic targets.

**Table 1:** Demographic and clinical features for patients with ICI-IA and ICI-treated controls. Patient details are provided in this table. ICI: immune checkpoint inhibitor; NSCLC: Non small cell lung cancer; GI: gastrointestinal; GU: genitourinary; CDAI: clinical disease activity index

Variable	ICI-IA (*N* = 80)	ICI-treated controls (*N* = 36)
**Age**	61.4 (13.8)	67 (7.9)
**Sex**	Female: 49 Male: 31	Female: 18 Male: 18
**Race**	White: 71 Black: 4 Asian: 4 Other: 1	White: 29 Black: 6 Native American: 1 Other: 1
**Cancer type**	Melanoma: 28 Lung cancer: 20 GI cancer: 11 GU cancer: 4 Breast Cancer: 4 Squamous cell: 4 Other: 9	NSCLC: 36
**ICI class**	PD-1/PD-L1: 54 CTLA-4: 2 Ipi/Nivo: 23 Nivo/Relatlimab: 1	PD-1/PD-L1: 34 Ipi/Nivo: 2
**Time ICI start to sample date (months)**	37.2 (32.0)	18 (19.7)
**IRAE (for controls)**	N/A	19 (52.8%)
**CDAI**	17.2 (11.1)	N/A
**CDAI Category**	Remission:2 Low: 21 Moderate: 35 High: 20	N/A
**Tenosynovitis present**	15 (19.2%)	N/A
**Enthesitis Y/N**	23 (29.9%)	N/A
**Required csDMARDs for ICI-IA**	29 (36.3%)	N/A
**Required biologics for ICI-IA**	19 (23.8%)	N/A
**On steroids at time of serum sample for ICI-IA**	30 (37.5%)	N/A
**Persistent IA > 6 months post ICI cessation **(*N* = **71)**	58 (81.7%)	N/A

**Table 2:** Serum cytokine levels stratified by IA severity. Median [IQR] cytokine levels per CDAI category for all patients. Krushkal Wallis test was performed to determine statitstical significance

Cytokine (pg/ml)	Low (*n* = 21)	Moderate (*n* = 35)	High (*n* = 20)	*p*-value*
**IFNγ**	23.4 [15.4, 67.8]	26.0 [10.2, 87.3]	33.4 [18.1, 85.0]	0.71
**IL-1**	0 [0, 1.5]	0 [0, 0.72]	0 [0, 0]	0.83
**IL-4**	0 [0, 0]	0 [0, 0.04]	0 [0, 0]	0.67
**IL-6**	9.6 [2.0, 22.4]	12.1 [4.2, 56.0]	58.4 [2.9, 100.2]	0.21
**IL-10**	3.0 [2.3, 3.5]	2.0 [1.2, 3.1]	3.0 [1.6, 5.0]	0.18
**IL-12p70**	0 [0, 0.10]	0 [0,0]	0 [0, 0]	0.67
**IL-17 A**	0 [0, 0]	0 [0, 3.6]	0 [0,0]	0.31
**TNFα**	7.1 [4.3, 9.3]	6.8 [5.1, 8.7]	8.9 [5.6, 13.3]	0.20
**VEGF-A**	791 [394, 1240]	1439 [817, 2002]	987 [476, 2946]	**0.02**

**Table 3:** Ordinal logistic regression for outcome of CDAI category. This table shows unadjusted and adjusted results for ordinal logistic regression. Analysis was adjusted for age, sex, number of other IrAEs, being on steroids at time of sample, and symptom duration

Cytokine	Unadjusted OR (95% CI)	*p*-value	Adjusted* OR (95% CI)	*p*-value	
**IFNγ**	0.999 (0.996, 1.002)	0.568	0.999 (0.996, 1.002)	0.436	
**IL-1**	0.893 (0.689, 1.158)	0.394	0.876 (0.666, 1.153)	0.346	
**IL-4**	1.124 (0.322, 3.922)	0.855	1.201 (0.326, 4.431)	0.783	
**IL-6**	1.0044 (1.00007, 1.0088)	**0.046**	1.0049 (1.00036, 1.010)	**0.035**	
**IL-10**	1.029 (0.978, 1.083)	0.272	1.041 (0.986, 1.098)	0.146	
**IL-12p70**	0.884 (0.741, 1.055)	0.173	0.889 (0.739, 1.069)	0.214	
**IL-17 A**	0.978 (0.928, 1.031)	0.412	0.985 (0.930, 1.043)	0.608	
**TNFα**	0.994(0.981, 1.007)	0.401	0.99 (0.98, 1.01)	0.263	
**VEGF-A**	1.0003 (1.0000, 1.0006)	**0.046**	1.0003 (1.0000, 1.0007)	**0.032**	

**Table 4:** Baseline cytokine levels stratified by TNF-I response. Comparison of baseline cytokine levels median [IQR] for those with and without arthritis response to TNF-inhibitor therapy. Mann-Whitney test was performed to determine statistical significance

Cytokine (pg/ml)	TNF-I response (*n* = 11)	No response (*n* = 5)	*p*-value*
**IL-6**	53.1 [3.2, 90.8]	2.1 [1.7, 2.1]	**0.03**
**TNFα**	8.0 [6.0, 12.5]	5.1 [4.3, 5.1]	**0.02**
**VEGF-A**	2246 [723, 5077]	1552 [1108,1958]	0.58

## Electronic supplementary material

Below is the link to the electronic supplementary material.

Supplementary Material 1

## Data Availability

No datasets were generated or analysed during the current study.
