# Supplementary material for: Higher levels of VEGF-A and TNFα in patients with immune checkpoint inhibitor-induced inflammatory arthritis
Source: Arthritis Res Ther. 2025 Apr 1;27:74. doi: 10.1186/s13075-025-03546-3 (PMC11959780; doi:10.1186/s13075-025-03546-3)
Supplement: Supplementary file 1 — Supplementary Material 1 [file 13075_2025_3546_MOESM1_ESM.docx]

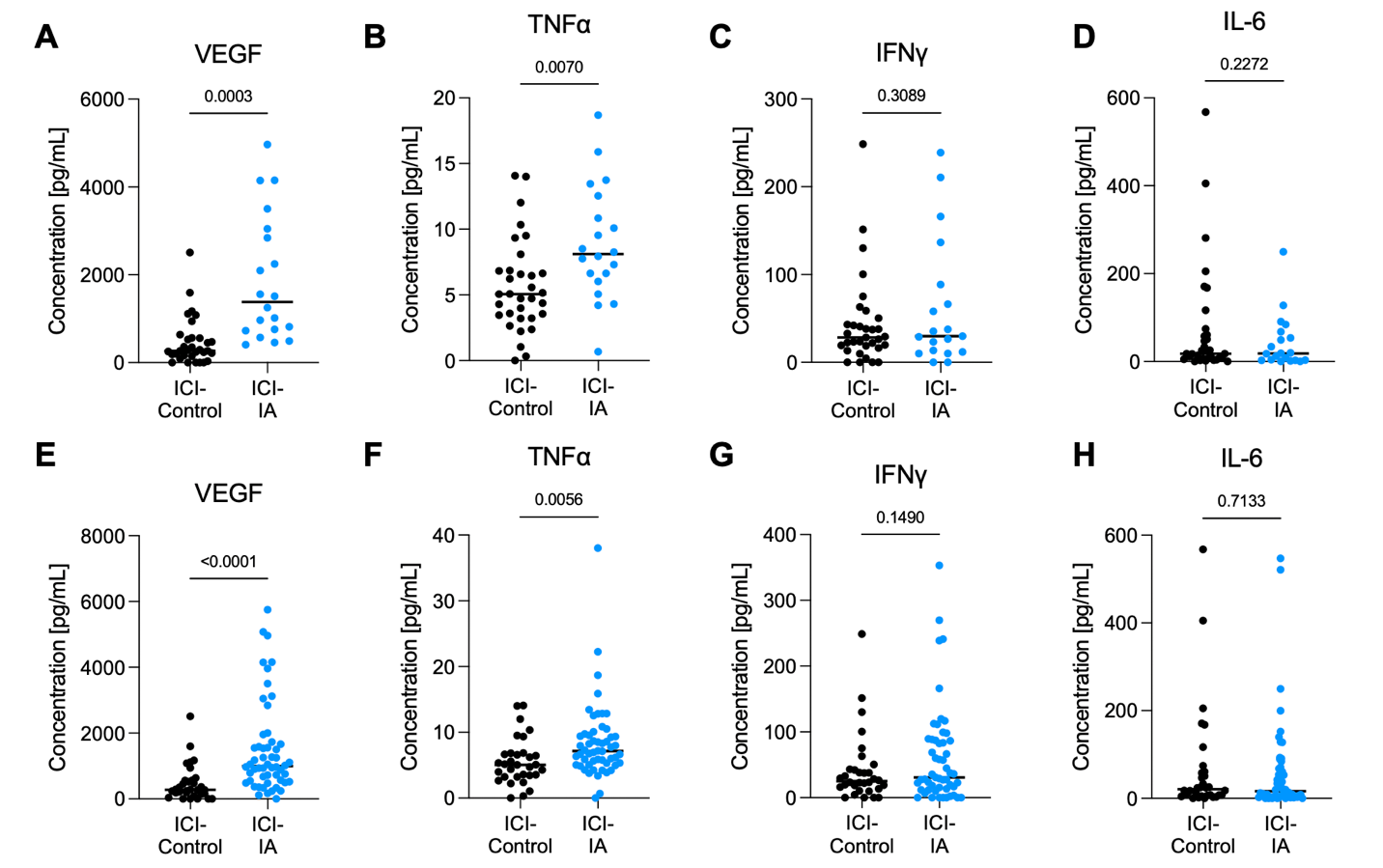


**Supplemental Figure 1. Serum cytokine levels of ICI-IA and ICI-Control cancer patients.** Abundance of (A,E) VEGF, (B,F) TNFα, (C,G) IFNγ, (D,H) IL-6 soluble factors in cancer patient serum treated with ICIs that developed ICI-IA (blue) versus patients treated with ICIs but did not develop ICI-IA (black). Patients with (A-D) lung cancer only or (E-H) monotherapy anti-PD-1/PD-L1 ICI treatment only are shown. Due to non-normal distribution of data, non-parametric Mann-Whitney U tests were run to determine significance.


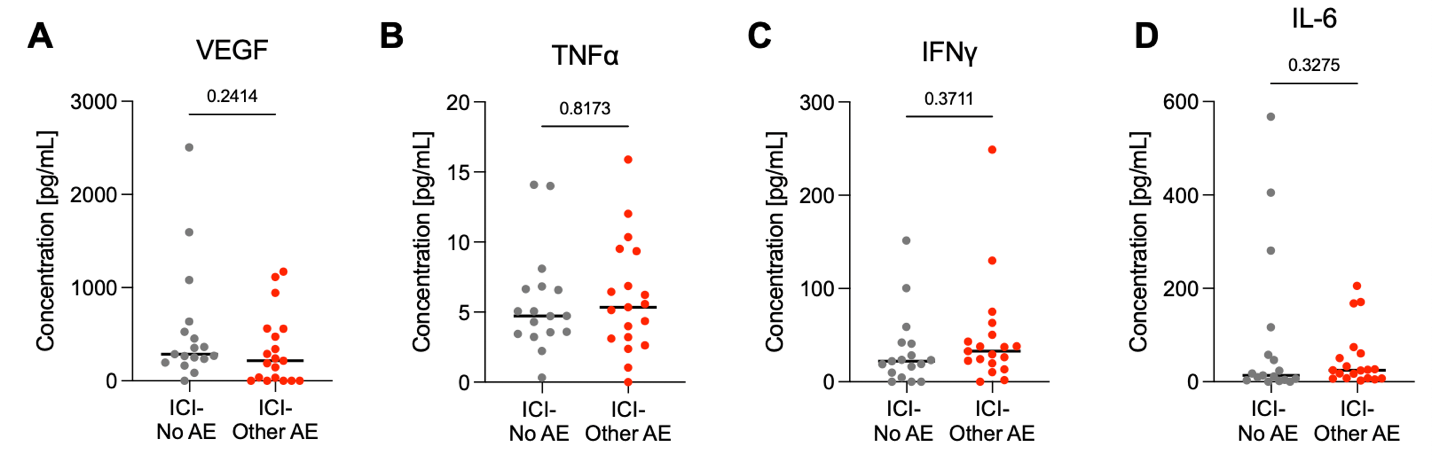


**Supplemental Figure 2**. **Serum cytokine levels of ICI-Control patients.** ICI-Control group serum levels of (A) VEGF, (B) TNFα, (C) IFNγ, (D) IL-6 separated by the development of other (not IA) adverse events (other AE, red) or no adverse event (no AE, grey). Due to non-normal distribution of data, statistical significance was determined via a non-parametric Mann-Whitney U test, and no significant differences were detected.

**Supplemental Table 1. Serum cytokine levels of ICI-IA and ICI-Control patients.** Abundance of soluble factors in cancer patient serum treated with ICIs that developed ICI-IA versus patients treated with ICIs but did not develop ICI-IA (control). ICI-IA patients are stratified by treatment with steroids or biologics, while ICI-control patients are stratified by diagnosis of another irAE. Median cytokine levels are listed with the interquartile range in brackets

| **Cytokine**  **(pg/ml)** | **All ICI-IA Patients (n=80)** | **ICI-IA w/o steroid/ biologic treatment (n=47)** | **ICI-IA with steroid/ biologic treatment (n=33)** | **All Control Patients (n=36)** | **Control-AE (n=19)** | **Control no-AE (n=17)** |
| --- | --- | --- | --- | --- | --- | --- |
| **VEGF-A** | 1050  [612.1, 1784] | 963  [554.7, 1712] | 1108  [791.6, 1840] | 268.1  [132.2, 534.4] | 216.4  [0, 559.4] | 286.3  [236.1, 526.1] |
| **TNFα** | 7.18  [4.93, 9.89] | 7.85  [5.45, 12.12] | 6.65  [4.25, 8.74] | 5.06  [3.40, 6.84] | 5.34  [3.21, 6.86] | 4.72  [3.56, 6.64] |
| **IFNγ** | 27.6  [13.64, 85.02] | 28.5  [14.03, 104.40] | 27.4  [10.12, 67.77] | 25.4  [15.58, 42.37] | 32.8  [22.71, 50.35] | 22.15  [9.98, 40.85] |
| **IL-6** | 13.4  [3.09, 60.18] | 21.0  [6.52, 65.83] | 8.59  [2.13, 55.98] | 20.75  [7.17, 58.55] | 24.69  [7.69, 60.96] | 13.66  [4.19, 57.75] |
| **IL-10** | 2.54  [1.59, 4.10] | 2.20  [1.20, 4.07] | 3.10  [1.91, 4.61] | 1.87  [1.23, 3.53] | 1.89  [0.973, 2.80] | 1.86  [1.40, 3.03] |
| **IL-17a** | 0  [0, 2.46] | 0  [0, 0] | 0  [0, 3.60] | 0  [0, 4.12] | 0  [0,2.76] | 0  [0, 5.14] |
| **IL-1α** | 0  [0, 0.36] | 0  [0, 1.24] | 0  [0, 0] | 0  [0, 3.08] | 2.04  [0, 3.02] | 0  [0, 0] |
| **IL-4** | 0  [0, 0] | 0  [0, 0] | 0  [0, 0] | 0  [0, 0] | 0  [0, 0] | 0  [0, 2.32] |
| **IL-12-p70** | 0  [0, 0] | 0  [0, 0] | 0  [0, 0.12] | 0  [0, 0] | 0  [0, 0.230] | 0  [0, 0] |

**Supplemental Table 2: Cytokine levels stratified by presence of tenosynovitis and enthesitis.** Comparison of baseline cytokine levels median [IQR]. Mann-Whitney test was performed to determine statistical significance.

| **Cytokine** | **Enthesitis**  **(n=23)** | **No Enthesitis**  **(n=54)** | **p-value** |  | **Tenosynvoitis**  **(n=15)** | **No Tenosynovitis**  **(n=63)** | **p-value** |
| --- | --- | --- | --- | --- | --- | --- | --- |
| **IFNγ** | 28.04  [18.93, 99.65] | 27.06  [10.11, 68.98] | 0.28 |  | 27.11  [6.74, 99.65] | 27.62  [13.73, 82.36] | 0.95 |
| **IL-1** | 0  [0, 1.75] | 0  [0, 0] | 0.28 |  | 0  [0, 0] | 0  [0, 1.27] | 0.013 |
| **IL-4** | 0  [0, 0] | 0  [0, 0] | 0.89 |  | 0  [0, 0] | 0  [0, 0] | 0.92 |
| **IL-6** | 30.40  [8.98, 73.36] | 10.64  [2.20, 59.66] | 0.18 |  | 6.02  [2.61, 92.75] | 18.80  [3.94, 60.70] | 0.35 |
| **IL-10** | 1.74  [1.15, 5.03] | 2.52  [1.73, 3.67] | 0.50 |  | 2.30  [1.36, 5.00] | 2.51  [1.60, 4.08] | 0.72 |
| **IL-12p70** | 0  [0, 0.86] | 0  [0, 0] | 0.72 |  | 0  [0, 0] | 0  [0, 0.23] | 0.07 |
| **IL-17A** | 0  [0, 3.60] | 0  [0, 2.89] | 0.86 |  | 0  [0, 2.89] | 0  [0, 3.60] | 0.22 |
| **TNFα** | 6.70  [3.87, 9.42] | 7.43  [5.06, 10.09] | 0.44 |  | 7.55  [4.25, 12.54] | 7.07  [4.97, 10.01] | 0.75 |
| **VEGF-Α** | 1049.56  [405.42, 1839.54] | 1019.30  [653.97,1653.50] | 0.81 |  | 570.29  [334.72, 1957.68] | 1164.32  [722.84, 1727.95] | 0.17 |

**Supplemental Table 3. Serum cytokine levels stratified by IA persistence.** Comparison of baseline cytokine levels median [IQR] for those with and without persistent IA. Only patients not on steroids at time of blood draw were included in this comparison. Mann-Whitney test was performed to determine statistical significance.

| **Cytokine (pg/ml)** | **Persistent IA**  **(n=35)** | **No Persistent IA**  **(n=9)** | **p-value*** |
| --- | --- | --- | --- |
| **IFNγ** | 26.7  [11.4, 119.9] | 82.4  [60.2, 117.0] | 0.12 |
| **IL-1** | 0  [0, 0.73] | 1.1  [0, 1.3] | 0.22 |
| **IL-4** | 0  [0, 0.17] | 0  [0, 0] | 0.61 |
| **IL-6** | 11.9  [2.1, 60.7] | 33.8  [30.4, 53.1] | 0.10 |
| **IL-10** | 2.0  [1.2, 3.5] | 2.3  [1.9, 4.1] | 0.42 |
| **IL=12p70** | 0  [0, 0] | 0  [0, 0.23] | 0.62 |
| **IL-17A** | 0  [0, 0] | 0  [0, 3.8] | 0.10 |
| **TNFα** | 7.3  [5.0, 12.6] | 8.3  [7.1, 12.8] | 0.33 |
| **VEGF-A** | 969  [494, 1584] | 730  [550, 1728] | 0.63 |

**Supplemental Table 4: Logistic regression evaluating ICI-IA persistence**. This table shows unadjusted and adjusted results for logistic regression. Analysis was adjusted for age, sex, number of other irAEs, being on steroids at time of sample, and symptom duration.

| **Cytokine** | **Unadjusted OR, 95% CI** | **p-value** | | **Adjusted OR, 95% CI** | **p-value** | | |
| --- | --- | --- | --- | --- | --- | --- | --- |
| **IL-6** | 1.001 (0.994, 1.008) | | 0.697 | 1.002 (0.994, 1.011) | | 0.593 |  |
| **IL-10** | 0.996 (0.936, 1.061) | | 0.909 | 0.981 (0.913, 1.054) | | 0.604 |  |
| **TNFα** | 1.008 (0.971, 1.043) | | 0.720 | 1.018 (0.949, 1.091) | | 0.619 |  |
| **VEGF-A** | 1.000 (0.999, 1.001) | | 0.427 | 1.000 (0.999,1001) | | 0.327 |  |
